# Atypical periprosthetic femoral fractures after arthroplasty for fracture are at high risk of complications

**DOI:** 10.1038/s41598-021-93574-1

**Published:** 2021-07-13

**Authors:** Tomonori Baba, Masataka Uchino, Hironori Ochi, Takuya Ikuta, Yoshitomo Saita, Hiroshi Hagino, Hiroaki Nonomiya, Seiya Jingushi, Takayuki Nakajima, Yasuhisa Ueda, Kaneko Kazuo

**Affiliations:** 1 Department of Orthopaedic Surgery, Juntendo University School of Medicine, 2-1-1 Hongo, Bunkyo-ku, Tokyo, Japan; 2 Department of Orthopaedic Surgery, Hakujikai Memorial Hospital, Tokyo, Japan; 3 Kumamoto Orthopedic Hospital, Kumamoto, Japan; 4 School of Health Science, Tottori University, Tottori, Japan; 5 Department of Orthopaedic Surgery, Shizuoka Red Cross Hospital, Shizuoka, Japan; 6 Department of Orthopaedic Surgery, Kyushu Rosai Hospital, Kitakyushu, Japan; 7 Department of Orthopaedic Surgery, Eastern Chiba Medical Center, Chiba, Japan; 8 Division of Orthopaedic Trauma, Sapporo Tokushukai Hospital, Sapporo, Japan

**Keywords:** Trauma, Fracture repair, Geriatrics, Diseases, Medical research, Risk factors, Health care, Therapeutics, Surgery

## Abstract

It is difficult to investigate clinical features in a single-center study because atypical periprosthetic femoral fracture (APFF) is rare. This study aims to perform a nationwide survey of APFF to investigate the characteristics of this fracture and compare the clinical outcome with that of typical periprosthetic femoral fracture (typical PFF). A nationwide survey was performed asking for cooperation from 183 councilors of the Japanese Society for Fracture Repair. The subjects were patients with APFF injured between 2008 and 2017. The control group was comprised of patients with typical PFF of our facility injured in the same period. A total of 43 patients met the APFF definition. The control group was comprised of 75 patients with typical PFF. The rate of bisphosphonate use was significantly higher in the APFFs group than in the typical PFF group (62.8% and 32%, *p* < 0.02). The rate of cemented stem was significantly higher in the APFFs group than in the typical PFF group (30.2% and 6.7%, *p* < 0.001). In the patients with arthroplasty for hip fracture, multivariable logistic regression analyses showed that APFF was an independent risk factor of complications following the initial management (Odds ratio 11.1, 95% confidence interval 1.05–117.2, *p* = 0.045). However, no significant association between PFF and APFF was observed in the patients with arthroplasty for other hip diseases. The risk of complications was higher in the APFF group than in the typical PFF group in the patients with arthroplasty for fracture. When AFPP after arthroplasty for the fracture is suspected, it may be necessary to add not only internal fixation with a normal plate but also some additional treatment.

## Introduction

Atypical femoral fracture (AFF) develops in the subtrochanteric region of the femur over the diaphysis. In this fracture, causative trauma is absent or transverse fracture is caused by slight trauma, showing characteristics not observed in typical fractures^1^. Periprosthetic femoral fracture (PFF) is excluded from AFF defined in the Task Force report of the American Society of Bone and Mineral Research (ASBMR). Long-term administration of bisphosphonate formulations (BPs) used to treat osteoporosis, use of steroid, and femoral curvature deformation are known as risks of onset of this fracture^2–4^. The bone union period protracts in many cases^5^, but it is possible to resume the activity level before fracture by surgical treatment in most cases, so that open reduction and internal fixation are recommended.

On the other hand, PFF is attracting attention as a fracture which will surely increase in the future with an increase in patients treated with total hip arthroplasty^6,7^. Since the femoral stem is positioned proximal to this fracture, it is necessary to devise internal fixation. In addition, the stem occupies the femoral medullary cavity and impedes medullary cavity blood flow, inhibiting bone union. There are diverse fracture types of PFF and difficulty in treatment of simple transverse fracture has been reported^8^. Case reports of PFF with a mechanism of injury and fracture morphology similar to those of AFF have recently been occasionally reported^9–12^ and we have also encountered several cases at our facility. Based on these findings, we consider that although it is excluded by ASBMR, PFF with characteristics similar to those of AFF surely exists. However, it is difficult to investigate clinical features of this fracture in detail in a single-center study because it is a relatively rare fracture.

The purpose of our study was to perform a nationwide survey of fracture around the stem with AFF morphology (atypical periprosthetic femoral fracture; APFF) present in a fewer number of cases to investigate the characteristics of this fracture and compare the clinical outcome with that of typical periprosthetic femoral fracture (typical PFF).

## Subjects and methods

The study was approved by hospital ethics committee of Juntendo University Hospital (17-293) and conducted in accordance with the Declaration of Helsinki. We applied Opt-out method to obtain consent for this study. A nationwide survey was performed asking for cooperation from 183 councilors of the Japanese Society for Fracture Repair.

**Figure 1:**
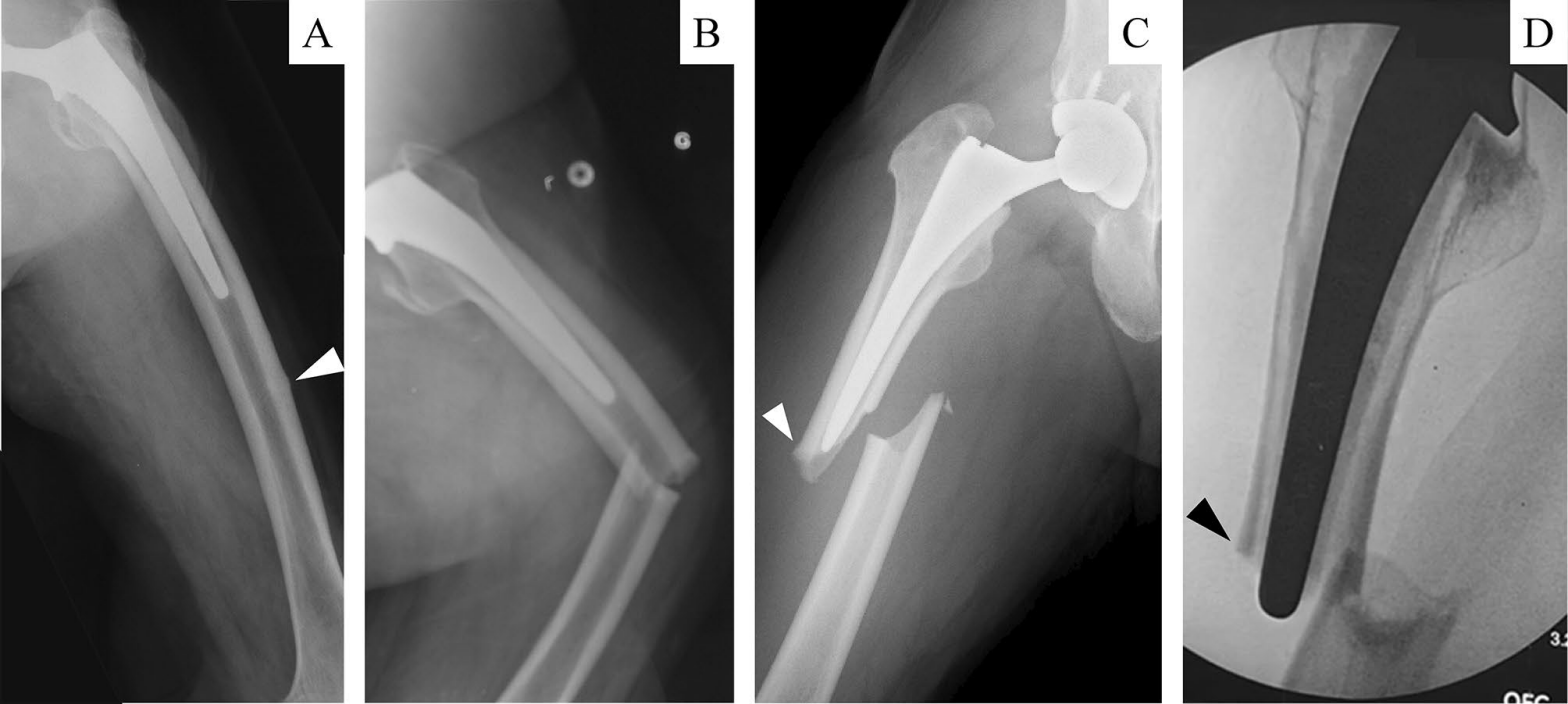
Atypical periprosthetic femoral fractures (APFF) (**A**) Incomplete fracture with uncemented stem inserted (arrowhead: localized periosteal reaction of the lateral cortex). (**B**) Progression from lateral cortex reaction to full periprosthetic femoral fracture. (**C**) APFF with uncemented stem inserted (arrowhead: localized periosteal reaction of the lateral cortex). (**D**) APFF with cemented stem inserted (arrowhead: localized periosteal reaction of the lateral cortex).

PFF with the AFF morphology was defined as a case meeting the following 2 conditions (Fig. 1): (1) An artificial hip joint implant is placed in the proximal femur. (2) Meeting at least 4 items of the major features following the definition of AFF established by JBMR in 2013 (periprosthetic femoral fractures are not excluded). The subjects were patients with APFF injured between January 1, 2008, and December 31, 2017. The control group was comprised of patients with typical PFF of our facility injured in the same period. The survey items were patient background (the age at the time of the first total hip arthroplasty, age at the time of fracture, sex, body mass index (BMI), existing disease, presence or absence of the use of cement in existing implant, and fracture region), history and period of the use of drugs for the treatment of osteoporosis, treatment method, and complications. Each group was confirmed by reviewing medical records.

### Statistical analysis

The patients’ characteristics were reported as means with standard deviations (SD), whereas qualitative variables are expressed as frequencies and percentages. The Student’s t-test was used for continuous data, and the Pearson chi-square test was applied for categorical variables to assess differences between groups. The adjusted odds ratio (OR) for complications following the initial treatment was estimated using a logistic regression model to determine the relationship between APFF and complications with adjustment for potential confounders such as cement or cementless stem and incomplete or complete fracture. Statistical analysis was performed using STATA statistical software (version 14.0; Stata Inc., College Station, TX, USA) and the level of significance was defined as a *p* value of < 0.05.

## Results

### Study participants and baseline characteristics

From 22 facilities to which the cooperating councilors belong, 43 patients met the APFF definition. The control group was comprised of 75 patients with typical PFF (Table 1). Patients’ characteristics were shown in Table 1. Regarding gender, 92.3% of APFFs occurred in women compared to 76.0% of typical PFFs (*p* = 0.049). There were no significant differences in the mean age of prior arthroplasty surgery and fracture between groups (8.5 ± 6.2 years and 7.8 ± 13.0 years, respectively, *p* = 0.74). The rate of bisphosphonate use was significantly higher in the APFFs group than in the typical PFF group (62.8% and 32%, respectively, *p* < 0.02). The rate of cemented stem was significantly higher in the APFFs group than in the typical PFF group (30.2% and 6.7%, respectively, *p* < 0.001). The rate of incomplete fracture was significantly higher in the APFFs group than in the typical PFF group (14% and none, respectively, *p* < 0.02). Complication rates were higher following the initial management of PAFFs (9/43, 20.9%) compared with typical PFFs (2/75, 2.7%) with a significant difference (Table 2).

**Table 1:** Characteristics of patients with atypical periprosthetic femoral fracture (APFF) and typical periprosthetic femoral fracture (PFF).

Variable	APFF (n = 43)	PFF (n = 75)	*p* value
Mean age, years (SD)	78.0 (8.4)	78.6 (10.0)	0.75
Mean age of prior arthroplasty surgery, years (SD)	70.5 (8.6)	71.9 (13.3)	0.56
Mean body mass index, kg/m2 (SD)	22.7 (4.2)	23.5 (4.4)	0.59
**Gender, n(%)**			0.049
Male	4 (7.6%)	18 (24%)	
Female	39 (92.3%)	57 (76%)	
**Diagnosis, n(%)**			0.01
Osteoarthritis	17 (39.5%)	13 (17.3%)	
Rheumatoid arthritis	2 (4.7%)	3 (4%)	
Osteonecrosis	3 (7.0%)	1 (1.3%)	
Femoral neck fracture	21 (48.8%)	58 (77.3%)	
**History of drugs for the treatment of osteoporosis, n(%)**	34 (79.1%)	40 (53.3%)	0.001
Bisphosphonate	27 (62.8%)	24 (32%)	0.002
Vitamin D	6 (16.3%)	14 (18.7%)	0.70
SERM	0	2 (2.6%)	0.28
Duration of bisphosphonate use, years(SD)	6.3 (3.1)	3.6 (3.4)	0.007

*SERM* selective estrogen receptor modulator.

**Table 2:** Implant and surgical characteristics of patients with atypical periprosthetic femoral fracture (APFF) and typical periprosthetic femoral fracture (PFF).

Variable	APFF (n = 43)	PFF (n = 75)	*p* value
**Type of hip arthroplasty, n(%)**			0.003
Hemiartroplasty	20(46.5%)	57(76%)	
Total hip arthroplasty	23(53.5%)	17(22.7%)	
Revision hip arthroplasty	0	1(1.3%)	
**Fixation of stem, n(%)**			0.001
Cemented	13(30.2%)	5(6.7%)	
Cementless	30(69.8%)	70(93.3%)	
**Fracture type, n(%)**			0.002
Complete	37(86%)	75(100%)	
Incomplete	6(14%)	0	
**Management, n(%)**			0.37
Nonoperative	1(2.3%)	4(5.3%)	
Fracture fixation	35(81.3%)	53(70.7%)	
Revision arthroplasty	7(16.3%)	18(24%)	
**Any complication, n(%)**	7(16.3%)	1(1.3%)	0.002
Nonunion/breakage of plate	6(14%)	0	
Varus deformity	1(2.3%)	1(1.3%)	

### Relationship between the APFF and complication

After adjustment for covariates, multivariable logistic regression analysis demonstrated that APFFs were significantly associated with complications (Odds ratio 17.5, 95% confidence interval 1.98–155.8, *p* = 0.010) (Table 3).

**Table 3:** Multivariable logistic regression analyses.

	Complication					
	Number of subjects	Number of complication (%)	OR [95% CI]	*p* value	Adjusted OR [95% CI]	*p* value
**All subjects**						
PFF	75	1(1.3%)	Ref	–	Ref	–
APFF	43	7 (16.3%)	14.3 [1.70–121.4]	0.014	17.5 [1.98–155.8]	0.010
**Hip fracture**						
PFF	58	1 (1.7%)	Ref	–	Ref	–
APFF	21	4(19.0%)	13.4 [1.40–128.1]	0.024	11.1 [1.05–117.2]	0.045
Other hip diseases*						
PFF	17	0(0%)	Ref	–	Ref	–
APFF	22	3(13.6%)	–		–	

*Osteoarthritis, rheumatoid arthritis, osteonecrosis.

### Subgroup analysis

Subgroup analysis was conducted using the aetiology of arthroplasty, which is considered a particularly different bone quality between hip fracture or the other hip diseases such as osteoarthritis rheumatoid arthritis and osteonecrosis. In the patients with hip fracture, multivariable logistic regression analyses showed that APFF was an independent risk factor of complications following the initial management (Odds ratio 11.1, 95% confidence interval 1.05–117.2, *p* = 0.045) (Table 3). No significant association between PFF and APFF was observed in the patients with other hip diseases (Table 3).

## Discussion

AFF is a transverse fracture in which the fracture line starts from the lateral cortical bone, localized hypertrophy is observed in the outer periosteum or endosteum of the lateral bone cortex of the fracture region, and no crush is present in the fracture region, showing a characteristic simple radiographic image. On the other hand, femoral neck fracture, intertrochanteric fracture continuous to subtrochanteric spiral fracture, bone tumor-associated pathological fracture, and periprosthetic fracture are excluded from the definition of AFF. However, of periprosthetic fracture patients, those with the AFF morphology have become occasionally reported from about 10 years ago^13^. Several case series have recently been reported^14–17^, but the number of cases was small (Table 4). The strength of our study is that we were able to investigate many cases by performing a national survey. On the other hand, the limitation of this study is that it was a retrospective study based on medical records; therefore, the treatment strategy for osteoporosis, the implants, and the internal fixation for APFF were all decided by the surgeon. Only surgeons who were board members of the Japanese Society of Fracture Repair participated in this survey. Since there are more opportunities to treat APFFs in the institutions with which these members are affiliated than other institutions, a survey in such institutions represents a present status survey of the characteristics in patients with APFF in Japan.

**Table 4:** Summary of the literature on atypical periprosthetic femoral fracture (APFF).

	Robinson et al.^14^	Lee et al.^15^	MacKenzie et al.^17^	Leclerc et al.^16^	Our study
Total	21	7	16	11	43
Gender, M:F, n(%)	2:19 (10%:90%)	0:7 (0%:100%)	2:14 (12.5%:87.5%)	2:9 (18.8%:81.2%)	4:39 (7.6%:92.3%)
Mean age, years (SD or range)	80	81.8 (5.6)	73.9 (44 to 88)	72.3 (11.6)	78.0 (8.4)
History of BPs use, n(%)	10(100%)	6 (85.7%)	13 (81%)	8 (72.7%)	27 (62.8%)
Duration of BPs use, years (SD or range)	> 2	4.4 (3.6)	5.5 (3.2 to 10.6)	Unknown	6.3 (3.1)
Fracture displaced:undisplaced, n(%)	Unknown	6:1 (85.7%:14.3%)	Unknown	8:3 (72.7%:27.3%)	37:6 (86%:14%)
Stem, cemented:cementless, n(%)	Mostly uncemented	2:5 (28.6%:71.4%)	16:0 (100%:0)	5 :3 (62.5%:37.5%)	13:30 (30.2%:69.8%)
Surgery (nonoperative:ORIF:revision)	No revision	Unknown	0:13:3 (0:81%:19%)	Unknown	1:35:7 (2.3%:81.4%:16.3%)
Duration of bone union	8mo	Unknown	Unknown	Unknown	10.8mo (7.6)
Any complication, n(%)	25%	Unknown	9 (56%)	Unknown	9 (21%)
Complications related to bone union, n(%)	Unknown	Unknown	6 (38%)	Unknown	7 (16.3%)

Our findings clarified that the period of the use of bisphosphonate (BPs) was significantly longer and the rate of the use was significantly higher in the APFF than typical PFF group. Long-term BPs administration increases the risk of atypical femoral fractures, so it is recommended to reassess the fracture risk 3 to 5 years after the start of administration^18^. If there is already a history of proximal femur fracture, the risk of the next fracture is classified as high, so it is recommended to continue administration of BPs. Our findings suggest that the use of BPs in arthroplasty after hip fracture is not desirable for long-term administration, and another osteoporosis drug, such as teriparatide, is more suitable. The rate (72.7–100%) of the use of BPs in case series of other APFFs was also high. In AFF, involvement of severely suppressed bone turnover (SSBT) induced by long-term use of BPs^19^ has been pointed out, suggesting that SSBT is also involved in APFF. On the other hand, the rate of the use of BPs was 62.8% in our study and this is low in comparison with that in other case series, clarifying the presence of an onset factor other than long-term use of BPs for the development of APFF. Involvement of femoral bowing-induced stress fracture is known as an onset factor of AFF^20^. Femoral bowing is larger in Japanese than in other races^21^, suggesting that the risk of stress fracture around the femoral diaphysis is high in Japanese. The fracture occurred more frequently in the diaphysis than in the subtrochanteric region in our APFF group, suggesting involvement of femoral bowing-induced stress fracture, similar to that in AFF. The presence of incomplete fracture (14%) not present in PFF demonstrated involvement of stress fracture. Furthermore, the rate of cemented stem was four times higher or more in the APFF group than the PFF group (7.1% vs. 30.2%). In the present study, the main reason for using a cemented stem depended on the surgeons. According to the Japanese registry, the use of the cemented stems is only 7% of all cases; hence, the proportion of cemented stem in APFF patients was very high. Since a cemented stem fills the medullary cavity crossing over the stem tip, stress may be concentrated around the tip in patients with femoral bowing. The incidence of typical PFF was higher by 3 times or more in the cementless than the cemented stem patients in a systematic review (0.31% vs. 1.03%)^22^. These findings suggest that cemented stem may be a risk factor for APFF. However, there was no significant difference in BPs utilization between cemented and cementless stems in our study.

The frequency of occurrence of postoperative complication of AFF has been reported to be higher than that in normal femoral fracture^23–25^. In AFF, the bone union period protracts compared with that of normal fracture^5^, so that many cases of postoperative complication are nonunion and breakage of the fixation material. In this study, the influence of BPs for the outcome after APFF was not investigated. In general, if BPs are administered and APFF occurs, administration will be discontinued. The fixation material for APFF uses a plate because the implant occupies the medullary cavity. A systematic literature review on the frequency of occurrence of postoperative complications of typical PFF reported that the frequency of nonunion was 3.9%; however, when a locking plate was used, it improved to 0.9%^26^. Regarding the incidence of complications of APFF, Robinson et al.^14^ reported that it was higher than that of AFF (12% vs. 25%, *p*= 0.013). However, there was no description concerning complications of bone union, the details were pneumonia, heart disease, pulmonary infarction, wound infection, and death, and details of the number of patients was unclear. On the other hand, Mackenzie et al.^17^ reported that the infection and reoperation rates were higher in APFF than in typical PFF, but no significant difference was noted in the frequency of nonunion or mechanical complication of fixation. However, the frequency of nonunion in APFF was 13% and that of mechanical complication of fixation was 25%, being not low. In our findings, the incidence of complication was clearly higher in APFF than in PFF (20.9% vs. 2.7%). In addition, APFF in patients with arthroplasty due to a fracture had an increased risk of complications: nonunion or plate breakage was found in six patients (14.0%) and varus malunion was found in one patient (2.3%), clarifying that bone union ability is low in APFF and a prolonged time is required for bone union. Solutions to reduce complications of APFF include the following measures: (1) Addition of bone graft^27^, (2) strengthening of the internal fixation method^28^, and (3) correction of alignment of the fracture region^29^. Regarding bone graft, although there is no clear evidence in AFF^30^, the fracture region may be in a state of SSBT, for which autologous bone graft can be sufficiently expected to enhance biological activity. In patients with strong femoral bowing, although anatomical reduction and internal fixation can be applied, tensile stress constantly acts on the convex side of bowing, i.e., fracture region. There is a risk of plate breakage occurring before bone union because the bone union period is long in APFF. Since the strength of internal fixation cannot be retained until bone union with a normal single plate alone, augmentation by strut allografting and double plating is necessary^29^. Double plating may be an option easier to accept for this fracture because facilities which can readily obtain strut allografting are limited in Japan. We propose correction of alignment by osteotomy of the bowing fracture region into an open edge shape as another option. Reduction of stress on the plate by correction of alignment can be expected. However, we do not yet have enough data to support the recommendations to prevent complications and believe that more rigorous validation is needed.

## Conclusion

The characteristics of 43 cases of APFF, the largest number of patients, were presented as a multicenter study. It was clarified that the rate of the use of BPs was high, similar to that in AFF, and the frequency of diaphyseal fracture was high. Since the risk of complication was higher in APFF than in typical PFF in the patients with arthroplasty for fracture, when APFF after arthroplasty for the fracture is suspected, it may be necessary to add not only internal fixation with a normal plate but also some additional treatment.
